# Protective activity of mRNA vaccines against ancestral and variant SARS-CoV-2 strains

**DOI:** 10.1126/scitranslmed.abm3302

**Published:** 2021-11-30

**Authors:** Baoling Ying, Bradley Whitener, Laura A. VanBlargan, Ahmed O. Hassan, Swathi Shrihari, Chieh-Yu Liang, Courtney E. Karl, Samantha Mackin, Rita E. Chen, Natasha M. Kafai, Samuel H. Wilks, Derek J. Smith, Juan Manuel Carreño, Gagandeep Singh, Florian Krammer, Andrea Carfi, Sayda M. Elbashir, Darin K. Edwards, Larissa B. Thackray, Michael S. Diamond

**Affiliations:** ^1^ Department of Medicine, Washington University School of Medicine, St. Louis, MO 63110, USA; ^2^ Department of Pathology & Immunology, Washington University School of Medicine, St. Louis, MO 63110, USA; ^3^ Department of Molecular Microbiology, Washington University School of Medicine, St. Louis, MO 63110, USA; ^4^ Center for Pathogen Evolution, Department of Zoology, University of Cambridge, Cambridge UK CB2 3EJ; ^5^ Department of Microbiology, Icahn School of Medicine at Mount Sinai, New York, NY 10029, USA.; ^6^ Moderna, Inc., Cambridge, MA 02139, USA; ^7^ The Andrew M. and Jane M. Bursky Center for Human Immunology and Immunotherapy Programs, Washington University School of Medicine. St. Louis, MO 63110, USA

## Abstract

Although mRNA vaccines encoding the spike protein of SARS-CoV-2 prevent COVID-19, the emergence of new viral variants jeopardize their efficacy. Here, we assessed the immunogenicity and protective activity of historical (mRNA-1273, designed for Wuhan-1 spike protein) or modified (mRNA-1273.351, designed for B.1.351 spike protein) Moderna mRNA vaccines in 129S2 and K18-hACE2 mice. Mice were immunized with either high-dose or low-dose formulations of the mRNA vaccines, where low-dose vaccination modeled suboptimal immune responses. Immunization with formulations at either dose induced neutralizing antibodies in serum against ancestral SARS-CoV-2 WA1/2020 and several virus variants, although serum titers were lower against the B.1.617.2 (Delta) virus. Protection against weight loss and lung pathology was observed with all high-dose vaccines against all viruses. However, low-dose formulations of the vaccines, which produced lower magnitude antibody and T cell responses, showed breakthrough lung infections with B.1.617.2 and development of pneumonia in K18-hACE2 mice. Thus, in individuals with reduced immunity following mRNA vaccination, breakthrough infection and disease may occur with some SARS-CoV-2 variants.

## Teaser

mRNA-1273-based vaccines protect against historical and variant SARS-CoV-2 strains in 129S2 and K18-hACE2 mice.

## INTRODUCTION

Severe acute respiratory syndrome coronavirus 2 (SARS-CoV-2) is the cause of the Coronavirus Disease 2019 (COVID-19). More than 256 million infections and 5.1 million deaths have been recorded worldwide (https://covid19.who.int) since the start of the pandemic. The extensive morbidity and mortality associated with the COVID-19 pandemic made the development of SARS-CoV-2 vaccines a global health priority. In a period of less than one year, several highly effective vaccines targeting the SARS-CoV-2 spike protein encompassing multiple platforms (lipid nanoparticle encapsulated mRNA, inactivated virion, or viral-vectored vaccine platforms (*1*)) gained Emergency Use Authorization or Food and Drug Administration approval and were deployed with hundreds of millions of doses given worldwide (https://covid19.who.int). The currently used vaccines were all designed against the spike protein of strains that were circulating early in the pandemic. In localities with high rates of vaccination, markedly reduced numbers of infections, hospitalizations, and deaths were initially observed.

Despite the success of COVID-19 vaccines and their potential for curtailing the pandemic, the continued evolution of more transmissible SARS-CoV-2 variants of concern (VOC) jeopardizes the efficacy of vaccination campaigns. These VOC include B.1.1.7 (Alpha), B.1.351 (Beta), B.1.1.28 (Gamma), and B.1.617.2 (Delta), all of which have substitutions in the spike protein (*2*). Experiments in cell culture suggest that neutralization by vaccine-induced serum is diminished against variants expressing mutations in the spike gene at positions L452, E484, and elsewhere (*3*–*8*). Moreover, viral-vectored (ChAdOx1 nCoV-19 and Ad26.CoV2) and protein nanoparticle (NVX-CoV2373)-based vaccines showed reduced activity (10 to 60%) against symptomatic infection caused by the B.1.351 variant in clinical trials in humans (*9*–*11*), whereas mRNA-based vaccines (such as BNT162b2) retained substantial (about 75%) efficacy against the B.1.351 variant in humans with almost complete protection against severe disease (*12*).

Immunization of humans with two 100 μg doses of the lipid nanoparticle-encapsulated mRNA-1273 vaccine encoding a proline-stabilized full-length SARS-CoV-2 spike glycoprotein corresponding to the historical Wuhan-Hu-1 virus conferred 94% efficacy against symptomatic COVID-19 in clinical trials performed in the United States (*13*). More recent data in non-human primates shows that vaccination with two doses of mRNA-1273 results in an effective immune response that controls upper and lower respiratory tract infection after challenge with the SARS-CoV-2 B.1.351 variant (*14*). As an alternative approach, several manufacturers have designed modified vaccines that target specific VOC, including B.1.351, for possible immunization or boosting. Indeed, a mRNA-1273.351 vaccine recently was generated, which encodes a proline stabilized full-length SARS-CoV-2 spike glycoprotein from the B.1.351 virus. Here, we evaluated the immunogenicity and protective activity of lipid-encapsulated mRNA-1273 and mRNA-1273.351 Moderna vaccines in the context of challenge of wild-type 129S2 and human ACE2 (hACE2) transgenic (K18-hACE2) mice with historical and emerging SARS-CoV-2 strains including several key VOC.

## RESULTS

**Fig. 1.**
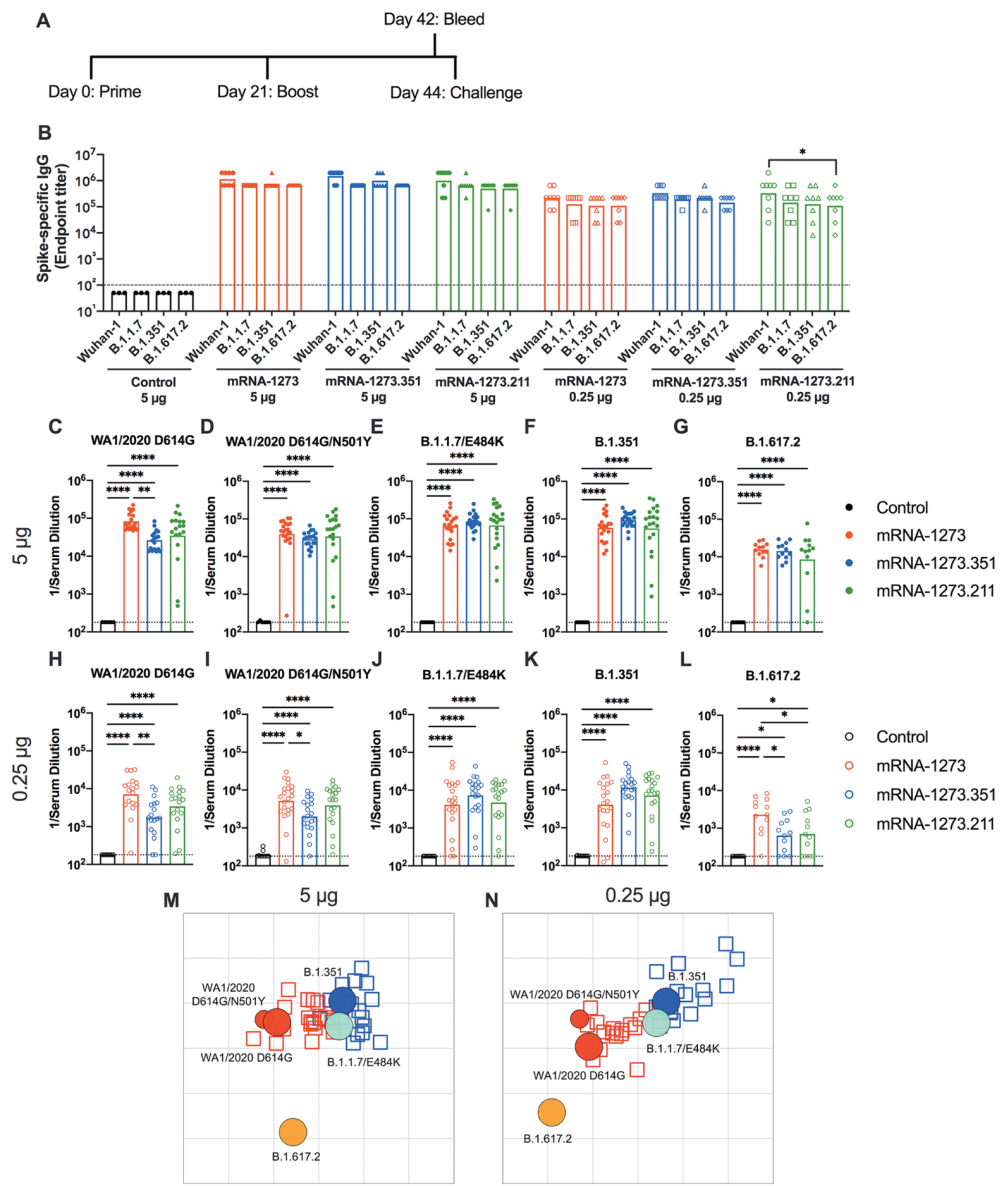
mRNA vaccines are immunogenic in 129S2 mice. Seven to nine-week-old female 129S2 mice were immunized and boosted with 5 or 0.25 μg of mRNA vaccines. (**A**) A scheme of immunizations, blood draw, and virus challenge is shown. (**B**) Serum anti-spike IgG responses were measured against indicated spike proteins at three weeks after booster immunization with mRNA vaccines (control, mRNA-1273, mRNA-1273.351, and mRNA-1273.211) (n = 3 (control vaccine) or 8 (spike vaccines), two experiments). Boxes illustrate mean values, and dotted line shows the limit of detection (LOD). Data were analyzed using a two-way ANOVA with Tukey’s post-test: *P < 0.05. (**C** to **L**). Serum neutralizing antibody responses three weeks after boosting were assessed by FRNT with WA1/2020 D614G (C and H), WA1/2020 D614G/N501Y (D and I), B.1.1.7/E484K (E and J), B.1.351 (F and K), or B.1.617.2 (G and L) in mice immunized with 5 (C to G) or 0.25 (H to L) μg of control (n = 6 to 10), mRNA-1273, mRNA-1273.351, or mRNA-1273.211 (n = 12 to 21) vaccines (two experiments). Boxes illustrate geometric mean values, dotted line shows LOD. Data were analyzed using a one-way ANOVA with Tukey’s post-test: *P < 0.05; **P < 0.01; ****P < 0.0001. (**M** and **N**) An antigenic map of serum samples from 129S2 mice is shown titrated against WA1/2020 D614G, WA1/2020 N501Y/D614G, B.1.1.7/E484K, B.1.351, and B.1.617.2. The maps show serum from mice that received 5 μg (M) or 0.25 μg (N) doses, respectively. Antigens (viruses) are shown as circles (WA1/2020 D614G: red, bigger circle, WA1/2020 N501Y/D614G: red, smaller circle, B.1.1.7/E484K: turquoise, B.1.351: blue, and B.1.617.2: orange), and serum samples are shown as squares (blue for mRNA-1273.351-induced serum and red for mRNA-1273-induced serum). The X and Y axes correspond to antigenic distance, with one grid line corresponding to a two-fold serum dilution in the neutralization assay. The antigens and serum samples are arranged on the map such that the distances between them best represent the distances measured in the neutralization assay.

**mRNA vaccines are immunogenic in 129S2 mice.** We first tested preclinical versions of the Moderna mRNA-1273 and mRNA-1273.351 vaccines encoding sequenced-optimized prefusion-stabilized spike proteins of Wuhan-1 and B.1.351, respectively, in immunocompetent 129S2 mice. These animals are permissive to infection by some SARS-CoV-2 variants (including B.1.1.7, B.1.1.28, and B.1.351) or mouse-adapted strains (*15*–*17*) that encode an N501Y mutation, which enables engagement of endogenous murine angiotensin converting enzyme 2 (ACE2) (*18*). Infection of 129S2 mice with SARS-CoV-2 results in mild to moderate lung infection and clinical disease with subsequent recovery (*15*, *17*). To assess the immunogenicity of the vaccines, groups of 7 to 9-week-old female 129S2 mice were immunized and boosted three weeks later by an intramuscular route with 5 μg (high) or 0.25 μg (low) doses of mRNA-1273, mRNA-1273.351, mRNA-1273.211 (1:1 mixture [total 5 or 0.25 μg] of mRNA-1273 and mRNA-1273.351), or a control untranslated mRNA (**Fig. 1A**); we included the mRNA-1273.211 mixture since it is being tested in humans (NCT04927065 (*19*)). A lower vaccine dose (0.25 μg) arm was included as a model for suboptimal responders and for evaluating correlates of protection, as we expected a greater frequency of breakthrough infections after SARS-CoV-2 challenge of this group. Serum samples were collected three weeks after boosting, and IgG responses against recombinant spike proteins of ancestral (Wuhan-1) or variant (B.1.1.7, B.1.351, or B.1.617.2) viruses (*20*) were evaluated by enzyme-linked immunosorbent assay (ELISA) (**Fig. 1B**). As expected, the control mRNA did not generate spike-specific IgG (values below the limit of detection), whereas antibody responses against the spike proteins from all other mRNA vaccines were robust. For the 5 μg dose, mean endpoint titers of serum ranged from 619,650 to 1,503,560 against the different spike proteins with little variation between the mRNA vaccines. For the 0.25 μg dose, approximately 5-fold lower serum IgG responses were observed with mean endpoint titers ranging from 126,900 to 382,725, again with little difference between the mRNA vaccines. Responses to different spike proteins for each vaccine generally were similar. Overall, both doses and all spike protein-based mRNA vaccines generated anti-spike protein IgG responses in 129S2 mice.

We characterized serum antibody responses functionally by assaying inhibition of SARS-CoV-2 infectivity using a focus-reduction neutralization test (FRNT) (*21*). We tested a panel of serum samples from each group of vaccinated mice against several fully-infectious SARS-CoV-2 strains, including an ancestral Washington strain with a single D614G substitution (WA1/2020 D614G) or one with both D614G and N501Y substitutions (WA1/2020 D614G/N501Y), a B.1.1.7 isolate encoding an E484K mutation (B.1.1.7/E484K), a B.1.351 isolate, and a B.1.617.2 isolate (**Fig. 1C to L**). Due to the limited amount of serum recovered from live animals, we started dilutions at 1:180. As expected, serum from all control mRNA-immunized mice did not inhibit infection of the SARS-CoV-2 strains (**Fig. 1C to L**). For the 5 μg dose, all three spike protein-based mRNA vaccines (mRNA-1273, mRNA-1273.351, and mRNA-1273.211) induced robust serum neutralizing antibody responses (**Fig. 1C to G**). In general, these titers were similar with the exception of about 4-fold lower geometric mean titers (GMTs) against WA1/2020 D614G and about 2-fold higher GMTs against B.1.351 induced by mRNA-1273.351 compared to the mRNA-1273 and mRNA-1273.211 vaccines. Lower neutralizing responses (about 4- to 5-fold) were seen against the B.1.617.2 strain by all three mRNA vaccines (**Fig. 1G**). For the 0.25 μg vaccine dose, we observed about 10-fold lower titers of serum neutralizing activity against each of the viruses (**Fig. 1H to L**). We also noted the following: (i) the mRNA-1273.351 vaccine induced lower titers of neutralizing antibody against WA1/2020 D614G and WA1/2020 D614G/N501Y than the mRNA-1273 vaccine (**Fig. 1H and I**); (ii) the mRNA-1273.211 mixture induced neutralizing antibodies that were equivalent to one of the two vaccine components; (iii) serum from mRNA-1273-vaccinated mice showed less reduction in neutralization against B.1.351 than anticipated based on prior studies in humans and C57BL6 mice (*6*, *7*) (**Fig. 1K**); and (iv) serum neutralizing antibody titers from all vaccinated mice were lower against B.1.617.2 than other strains, although responses from animals administered mRNA-1273 were slightly higher (**Fig. 1L**). Overall, these differences were visualized best in a comparative analysis of the inhibitory activity of each serum sample for the 5 μg (**fig. S1A to C**) and 0.25 μg (**fig. S1D to F**) doses.

Using the neutralization data from mRNA vaccinated 129S2 mice, we created antigenic maps to visualize the relationships between the WA1/2020 D614G, WA1/2020 D614G/N501Y, B.1.1.7/E484K, B.1.351, and B.1.617.2 SARS-CoV-2 strains (**Fig. 1M and N**). Neutralization titers obtained after 5 or 0.25 μg dosing with mRNA-1273 and mRNA-1273.351 vaccines were used to position the serum relative to each virus using antigenic cartography (a modification of multidimensional scaling for binding assay data), such that higher neutralization titers are represented by shorter distances between serum and the virus. Each gridline, or antigenic unit, of the map corresponds to a 2-fold difference in neutralization titer of a given virus. Three antigen clusters were observed: (i) WA1/2020 D614G and WA1/2020 D614G/N501Y grouped together; (ii) viruses containing E484K mutations (B.1.1.7/E484K and B.1.351) had a similar antigenic position; and (iii) B.1.617.2 was the most distant antigenically, which is consistent with the lower serum neutralization titers induced by all of the mRNA vaccines against this VOC. In addition to providing a visual representation of the antigenic relationships observed in **Fig. 1C to L**, the antigenic maps also show some differences between the 5 and 0.25 μg groups, in particular the movement of the B.1.617.2 virus leftwards. In the 5 μg dosing, the B.1.617.2 strain escaped serum antibodies from both mRNA-1273 and mRNA-1273.351 vaccines similarly (roughly equidistant position), whereas in the lower 0.25 μg dosing, the leftwards position of B.1.617.2 indicates a greater distance and antigenic escape from serum generated by the mRNA-1273.351 than the mRNA-1273 vaccine.

**Fig. 2.**
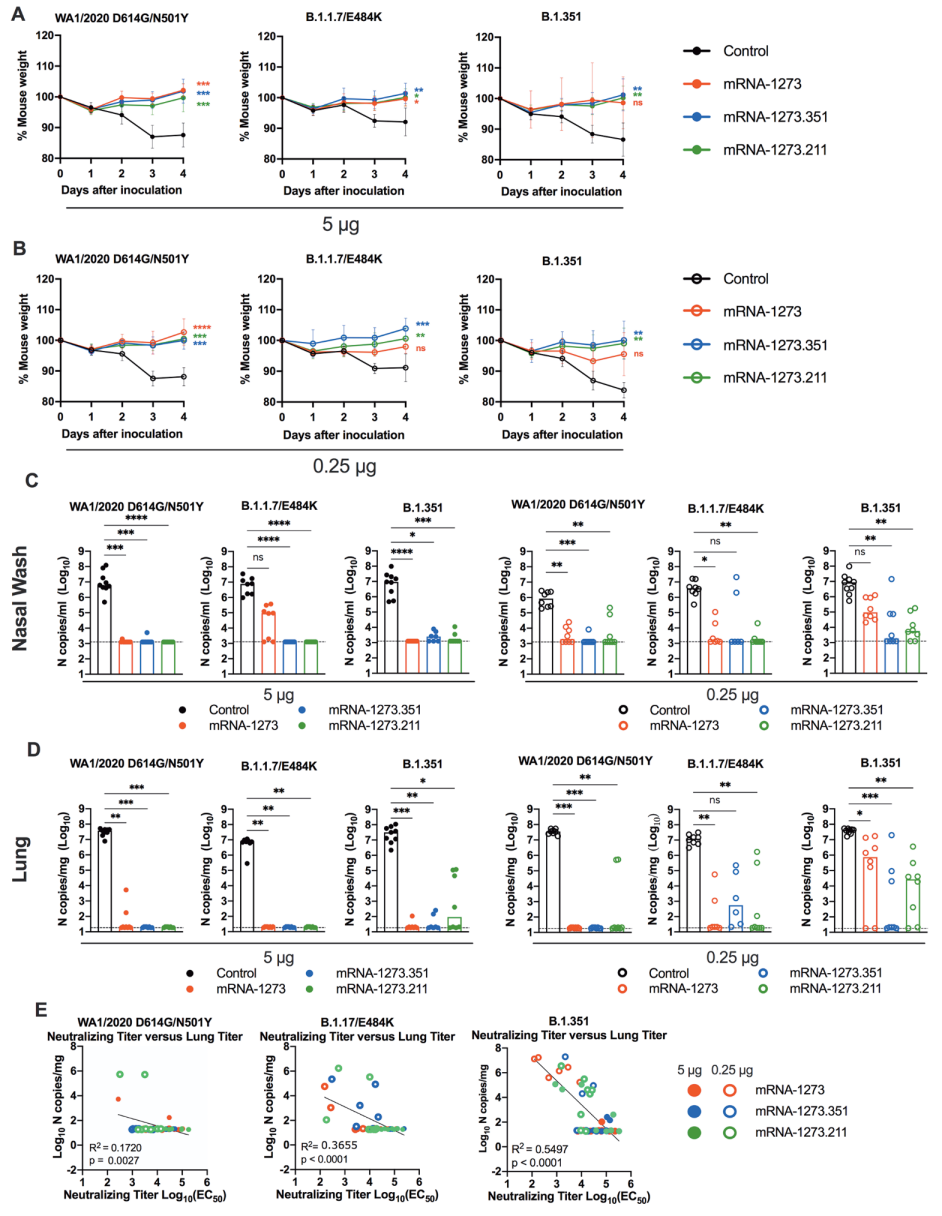
mRNA vaccination confers protection against SARS-CoV-2 infection after in 129S2 mice. Seven to nine-week-old female 129S2 mice were immunized and boosted with 5 or 0.25 μg of mRNA vaccines as described in Fig. 1A. Three weeks after boosting, mice were challenged by intranasal inoculation with 10^5^ focus-forming units (FFU) of WA1/2020 N501Y/D614G, B.1.1.7/E484K, or B.1.351. (**A** and **B**) Body weight change was measured over time. Data shown is the mean ± SEM (n = 6 to 9 mice per group, two experiments). Data were analyzed by a one-way ANOVA of the area under the curve from 2 to 4 dpi with Dunnett’s post-test; comparison to control immunized group: ns, not significant; *P < 0.05; **P < 0.01; ***P < 0.001; ****P < 0.0001). (**C** and **D**) Viral burden at 4 dpi in the nasal washes (C) and lungs (D) was assessed by qRT-PCR of the *N* gene after challenge of immunized mice (n = 6 to 8 mice per group, two experiments). Boxes illustrate median values, and dotted line shows LOD. Data were analyzed by a one-way Kruskal-Wallis ANOVA with Dunn’s post-test; comparison among all immunization groups: ns, not significant; *P < 0.05; **P < 0.01; ***P < 0.001; ****P < 0.0001). (**E**) Correlation analyses are shown comparing serum neutralizing antibody concentrations three weeks after boosting plotted against lung viral titer (4 dpi) in 129S2 mice after challenge with the indicated SARS-CoV-2 strain. EC_50_, half maximal effective concentration. Pearson’s correlation P and R2 values are indicated as insets. Closed symbols, 5 μg vaccine dose; open symbols, 0.25 μg vaccine dose.

**mRNA vaccines confer protection against SARS-CoV-2 in 129S2 mice.** We tested the protective activity of the different mRNA vaccines in 129S2 mice. Three weeks after boosting, mice were challenged by an intranasal route with WA1/2020 N501Y/D614G, B.1.1.7/E484K, or B.1.351. The WA1/2020 D614G and B.1.617.2 viruses were not used for challenge in this model since they lack the mouse-adapting N501Y substitution and cannot infect conventional laboratory mice (*16*). Compared to the control mRNA vaccine, the 5 μg or 0.25 μg doses of mRNA-1273, mRNA-1273.351, or mRNA-1273.211 vaccines all prevented weight loss between 2 and 4 days post-infection (dpi), although protection for some mice was not observed following immunization with the mRNA-1273 vaccine and challenge with B.1.351 or B.1.1.7/E484K viruses (**Fig. 2A and B**).

At 4 dpi, mice were euthanized, and nasal washes, lungs, and spleen were collected for viral burden analysis. In the nasal washes or lungs from control mRNA-vaccinated 129S2 mice, high amounts (about 10^7^ copies of *N* per mL or mg) of viral RNA were measured after challenge with WA1/2020 N501Y/D614G, B.1.1.7/E484K, or B.1.351 (**Fig. 2C and D****).** Lower amounts of SARS-CoV-2 RNA (about 10^2^ to 10^4^ copies of *N* per mg) were measured in the spleen (**fig. S2A**). In general, the mRNA-1273, mRNA-1273.351, and the mRNA-1273.211 vaccines conferred robust protection against infection in nasal washes, lungs, and spleens by the challenge SARS-CoV-2 strains, although some breakthrough was noted. After the 5 μg dose immunization with mRNA-1273, moderate B.1.1.7/E484K infection was detected in nasal washes in 5 of 8 mice, although viral RNA was absent from the lungs. Three of 8 mice immunized with the mRNA-1273.211 mixture also showed breakthrough in the lungs, albeit at greater than 100-fold lower quantities than the control vaccine. In comparison, the 5 μg dose of mRNA-1273.351 was protective in the nasal wash and lungs against all viruses, with little, if any, viral RNA measured.

As expected, the 0.25 μg dose of the mRNA vaccines showed less protective efficacy against SARS-CoV-2 challenge. Protection was conferred by the 0.25 μg dose against WA1/2020 N501Y/D614G and B.1.1.7/E484K challenge in the nasal washes at 4 dpi, except for the mRNA-1273.351 vaccine against B.1.1.7/E484K challenge (**Fig. 2C**). In comparison, after B.1.351 challenge, 8 of 8 mice immunized with mRNA-1273 showed viral RNA in nasal washes, with 3 of 8 showing amounts that approached those seen in control-vaccinated mice. Protection was generated against B.1.351 by mRNA-1273.351 or the mRNA-1273.211 mixture vaccines, although breakthrough infections were detected. In the lungs, protection against infection with WA1/2020 N501Y/D614G was generated by all three mRNA vaccines (**Fig. 2D**). However, some infection was seen after B.1.1.7/E484K or B.1.351 challenge especially with the 0.25 μg dose vaccine formulations. For example, 6 of 8 mice immunized with 0.25 μg of mRNA-1273 had moderate to high amounts of B.1.351 viral RNA in their lungs at 4 dpi.

We assessed for correlations between vaccine-induced neutralizing antibody titers and protection against SARS-CoV-2 infection in the lung after virus challenge. Serum titers of neutralizing antibodies exhibited an inverse association with quantities of SARS-CoV-2 RNA in the lung (**Fig. 2E**) with a minimum neutralizing titer of approximately 5,000 required to prevent infection in the lung at 4 dpi. Most of the breakthrough infections occurred with the B.1.351 challenge at the 0.25 μg dose of vaccines. For reasons that remains unclear, the threshold for complete protection in the lung after challenge with WA1/2020 N501Y/D614G was lower (2 to 7-fold) than against the other viruses. Moreover, when we compared body weight change at 4 dpi with neutralizing titers, only animals challenged with B.1.351 showed a linear correlation (**fig. S2B**), possibly because of the greater number of breakthrough infections in this group.

**Fig. 3.**
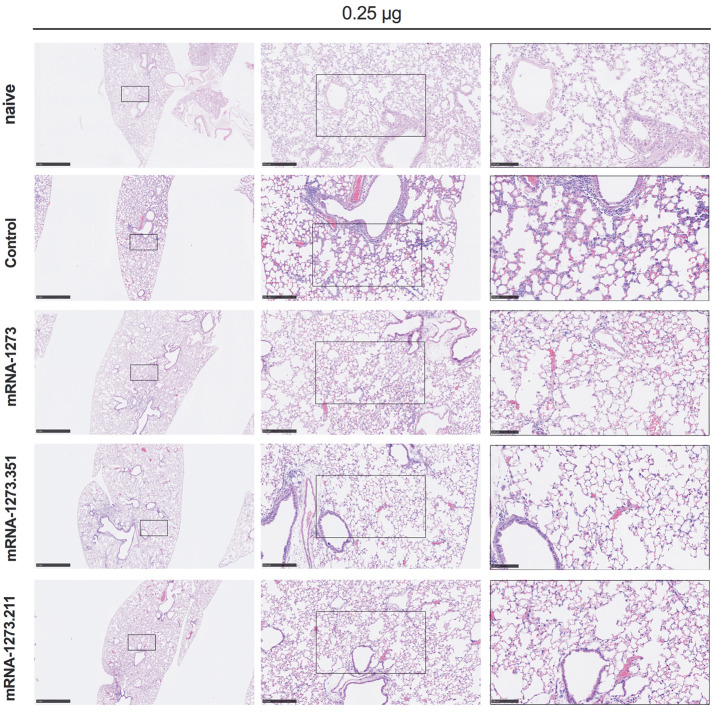
Vaccination mediates protection against lung pathology in 129S2 mice. Seven to nine-week-old female 129S2 mice were immunized, boosted with 0.25 μg of mRNA vaccines (control, mRNA-1273, mRNA-1273.351, or mRNA-1273.211), and challenged with B.1.351 as described in Fig. 2. Hematoxylin and eosin staining of lung sections harvested at 4 dpi or from a mock-infected (naïve) animal are shown. Images show low- (left; scale bars, 1 mm), medium- (middle; scale bars, 250 μm) and high-power (right; scale bars, 100 μm) magnification. Representative images are shown from n = 2 per group.

We also assessed the effect of the mRNA vaccines on lung disease at 4 dpi in129S2 mice. For these studies, we analyzed lung sections from the group of mice that received the lower 0.25 μg vaccine dose and the B.1.351 challenge virus, as this combination resulted in the greatest number of breakthrough infections. As expected, mice immunized with the control mRNA vaccine and challenged with B.1.351 developed mild pneumonia characterized by immune cell accumulation in perivascular and alveolar locations, vascular congestion, and interstitial edema. In contrast, animals immunized with mRNA-1273, mRNA-1273.351, or mRNA-1273.211 vaccines did not show these pathological changes (**Fig. 3**). Thus, immunization with even the low dose of the mRNA vaccines was sufficient to mitigate SARS-CoV-2-induced lung injury in immunocompetent 129S2 mice challenged with some VOC.

**Fig. 4.**
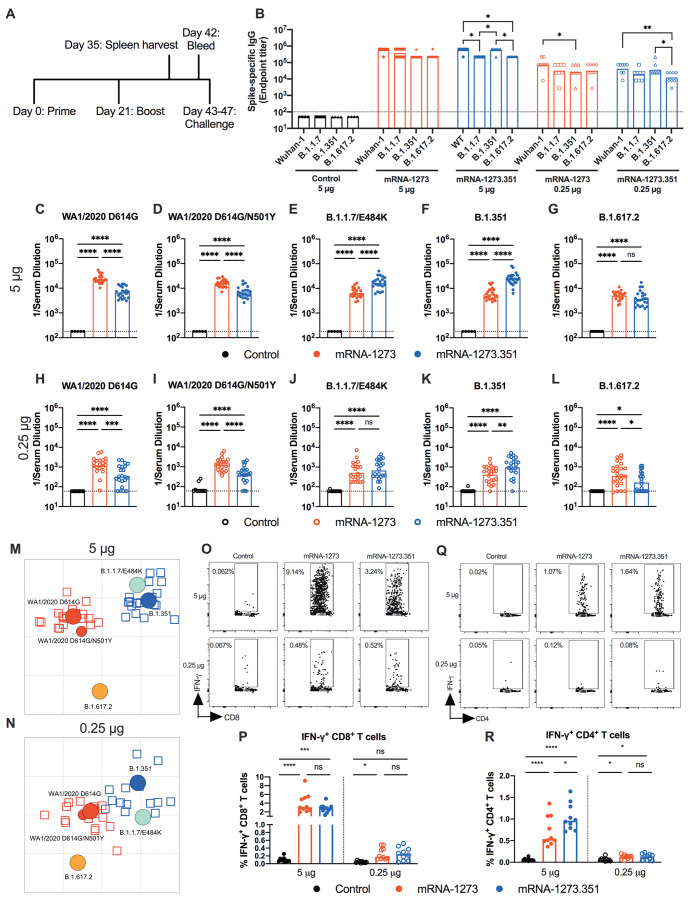
mRNA vaccines are immunogenic in K18-hACE2 transgenic mice. Seven-week-old female K18-hACE2 mice were immunized and boosted with 5 or 0.25 μg of mRNA vaccines. (**A**). The scheme shows timing of immunizations, spleen harvest, blood draw, and virus challenge. (**B**) Serum anti-spike IgG responses against indicated spike proteins were measured at three weeks after booster immunization with mRNA vaccines (n = 3 (control vaccine) or 8 (spike vaccines), two experiments). Boxes illustrate mean values, and dotted line shows the LOD. Data were analyzed by a two-way ANOVA with Tukey’s post-test: *P < 0.05; **P < 0.01). (**C** to **L**) Serum neutralizing antibody responses were measured at three weeks after boosting by FRNT with WA1/2020 D614G (C and H), WA1/2020 D614G/N501Y (D and I), B.1.1.7/E484K (E and J), B.1.351 (F and K), or B.1.617.2 (G and L) in mice immunized with 5 (C to G) or 0.25 (H to L) μg of control (n = 5 to 10), mRNA-1273 (n = 20 to 24), and mRNA-1273.351 (n = 21 to 24) vaccines (two experiments). Boxes illustrate geometric mean values, and dotted line shows LOD. Data were analyzed by a one-way ANOVA with Tukey’s post-test: ns, not significant; *P < 0.05; **P < 0.01; ***P < 0.001; ****P < 0.0001). (**M** and **N**) Antigenic maps are shown for serum samples from K18-hACE2 mice titrated against WA1/2020 D614G, WA1/2020 N501Y/D614G, B.1.1.7/E484K, B.1.351, and B.1.617.2. The maps show serum samples from immunized mice that received 5 μg (M) or 0.25 μg (N) doses, respectively, with symbol details described in Fig. 1. (**O** to **R**) CD8^+^ (O and P) and CD4^+^ (Q and R) T cell responses were measured in K18-hACE2 mice at day 35, two weeks after booster immunization with mRNA vaccines (n = 10 for each group, two experiments). Representative flow cytometry plots (O and Q) and quantification of IFN-γ responses (P and R) are shown. Boxes illustrate median values, one-way ANOVA with Tukey’s post-test: ns, not significant; *, P < 0.05; **, P > 0.01; ***, P < 0.001; **** P < 0.0001).

**mRNA vaccines are immunogenic in K18-hACE2 transgenic mice.** We next evaluated the mRNA-1273 and mRNA-1273.351 vaccines in K18-hACE2 transgenic mice, which are highly susceptible to severe infection and disease after intranasal inoculation by many SARS-CoV-2 strains (*22*) including isolates containing or lacking mouse-adapting mutations, such as N501Y (*17*). Due to a limited availability of K18-hACE2 mice and the need to test two control viruses (WA1/2021 D614G and WA1/2021 D614G/N501Y), we tested mRNA-1273 and mRNA-1273.351 but not the mRNA-1273.211 mixture vaccine. Groups of 7-week-old female K18-hACE2 mice were immunized and boosted three weeks later by intramuscular route with 5 or 0.25 μg doses of mRNA-1273, mRNA-1273.351, or control mRNA vaccine (**Fig. 4A**). Serum samples were collected three weeks after boosting, and IgG responses against recombinant spike proteins (Wuhan-1, B.1.1.7, B.1.351, or B.1.617.2) were evaluated by ELISA (**Fig. 4B**). Antibody responses against the different spike proteins were robust although slightly lower (about 2 to 3-fold) than that seen in 129S2 mice (**Fig. 1B**). Serum mean endpoint IgG titers ranged from 218,700 to 1,601,425 against the different spike proteins with little variation observed with the 5 μg doses of different mRNA vaccines. For the 0.25 μg dose, lower (about 6 to 10-fold) IgG titers were measured (24,300 to 101,250) with little difference between the mRNA-1273 and mRNA-1273.351 vaccines. Although the IgG titers against the B.1.617.2 spike protein were reduced slightly compared to the other SARS-CoV-2 spike proteins, in general, robust antibody responses were detected in K18-hACE2 mice.

We performed FRNTs to assess the neutralizing activity of pre-challenge serum against WA1/2020 D614G, WA1/2020 D614G/N501Y, B.1.1.7/E484K, B.1.351, and B.1.617.2 SARS-CoV-2 strains. Because of the limited amount of serum recovered from K18-hACE2 mice, we initially started dilutions at 1:180. As expected, serum from all control mRNA-immunized mice did not inhibit infection of the SARS-CoV-2 strains (**Fig. 4C to L**). In general, neutralizing antibody titers induced by 5 or 0.25 μg mRNA vaccine dosing were lower (about 3 to 6-fold) in immunized K18-hACE2 than from 129S2 mice. For the 5 μg dose, although both mRNA-1273 and mRNA-1273.351 vaccines induced robust serum neutralizing antibody responses, we observed the following (**Fig. 4C to G** **and fig. S3**): (i) the mRNA-1273.351 vaccine induced lower titers of neutralizing antibody against WA1/2020 D614G and WA1/2020 D614G/N501Y than the mRNA-1273 vaccine (**Fig. 4C and D**); (ii) a reciprocal pattern was observed against viruses containing E484K mutations. The mRNA-1273.351 vaccine induced higher titers of neutralizing antibody against B.1.1.7/E484K and B.1.351 than the mRNA-1273 vaccine (**Fig. 4E and F**); and (iii) no differences in neutralizing activity were observed with the mRNA-1273 and mRNA-1273.351 vaccines against the B.1.617.2 strain. Although responses were elevated, they were lower than against other strains (**Fig. 4G**). Similar patterns were observed for the 0.25 μg dose (**Fig. 4H to L**), although about 10-fold lower titers of neutralizing activity were induced by each vaccine against each of the viruses. Because of this, we started our dilution series at 1:60 for serum derived from animals immunized with the 0.25 μg dose of mRNA vaccines. In general, the pattern of neutralization paralleled results with the higher dose, with the mRNA-1273 vaccine performing better against historical WA1/2020 and WA1/2020 D614G/N501Y viruses (**Fig. 4H to I**). However, serum from mice vaccinated with mRNA-1273 or mRNA-1273.351 vaccines neutralized B.1.617.2 less efficiently (**Fig. 4L**), with several data points at the limit of detection (1:60: mRNA-1273, 4 of 24; mRNA-1273.351, 9 of 24); nonetheless, responses induced by mRNA-1273 against B.1.617.2 were slightly greater than those of mRNA-1273.351. A comparative analysis of the inhibitory activity of each serum sample for the 5 μg (**fig. S3A and B**) and 0.25 μg (**fig. S3C and D**) doses visually showed these differences, as serum induced by the mRNA-1273 vaccine consistently showed less neutralizing activity against B.1.1.7/E484K, B.1.351, and B.1.617.2, whereas serum from mRNA-1273.351-vaccinated mice had greater inhibitory activity against B.1.351 and B.1.1.7/E484K.

We used the neutralization data from mRNA-vaccinated K18-hACE2 mice to generate maps defining the antigenic relationships between WA1/2020 D614G, WA1/2020 D614G/N501Y, B.1.1.7/E484K, B.1.351, and B.1.617.2 SARS-CoV-2 strains (**Fig. 4M and N**). Serum obtained after 5 or 0.25 μg dosing with mRNA-1273 or mRNA-1273.351 vaccines was analyzed against the indicated viruses, and each antigenic unit corresponded to a 2-fold difference in neutralization titer of a given virus. The results were remarkably similar to that seen with 129S2 vaccinated mice (**Fig. 1M and N**): (i) WA1/2020 D614G and WA1/2020 D614G/N501Y grouped together; (ii) B.1.1.7/E484K and B.1.351 viruses, which contain E484K mutations, grouped near each other; and (iii) B.1.617.2 localized to a separate antigenic group. The antigenic maps visually represent some of the differences and similarities in neutralizing reactivity patterns seen in the 5 and 0.25 μg groups (**Fig. 4C to L**). As was seen in 129S2 mice (**Fig. 1M and N**), the B.1.617.2 virus moved leftward in the 0.25 μg group (**Fig. 4M and N**); thus, antigenic escape from mRNA-1273.351-induced serum appeared increased relative to mRNA-1273 serum, especially in the lower 0.25 μg dose group. We also note the movement in position of the B.1.1.7/E484K virus, a finding not seen in the 129S2 mice. In the 5-μg dose (**Fig. 4M**) it is positioned close to the B.1.351 virus and mRNA-1273.351 induced serum, since the B.1.1.7/E484K shows greater antigenic escape against serum generated from the mRNA-1273 than mRNA-1273.351 vaccine (**Fig. 4E**). In the 0.25 μg dose group, however, the B.1.1.7/E848K strain moves to a location more equidistant from both serum groups, suggesting more escape from neutralization by mRNA-1273.351-induced serum (**Fig. 4J**).

We also examined T cell responses in mRNA-vaccinated K18-hACE2 mice two weeks after boosting (**Fig. O to R**) using H-2^b^ restricted immunodominant peptides in the spike protein for CD8^+^ and CD4^+^ T cells. After peptide stimulation ex vivo and staining for intracellular interferon (IFN)-γ production, we detected a robust CD8^+^ T cell (2 to 4 percent positive) response in the spleens of animals immunized with 5 μg of the mRNA-1273 or mRNA-1273.351 vaccines (**Fig. 4O and P**). The response was approximately 10-fold lower in animals immunized with the 0.25 μg dose. Although we also detected a spike protein-specific CD4^+^ T cell response after immunization (0.5 to 1.5 percent positive) with the 5 μg dose of mRNA-1273 or mRNA-1273.351 vaccines, it was lower in magnitude (**Fig. 4Q and R**). Moreover, the low 0.25 μg dose mRNA-1273 or mRNA-1273.351 vaccines induced CD4^+^ T cell responses that were barely greater than the control mRNA vaccine.

**Fig. 5.**
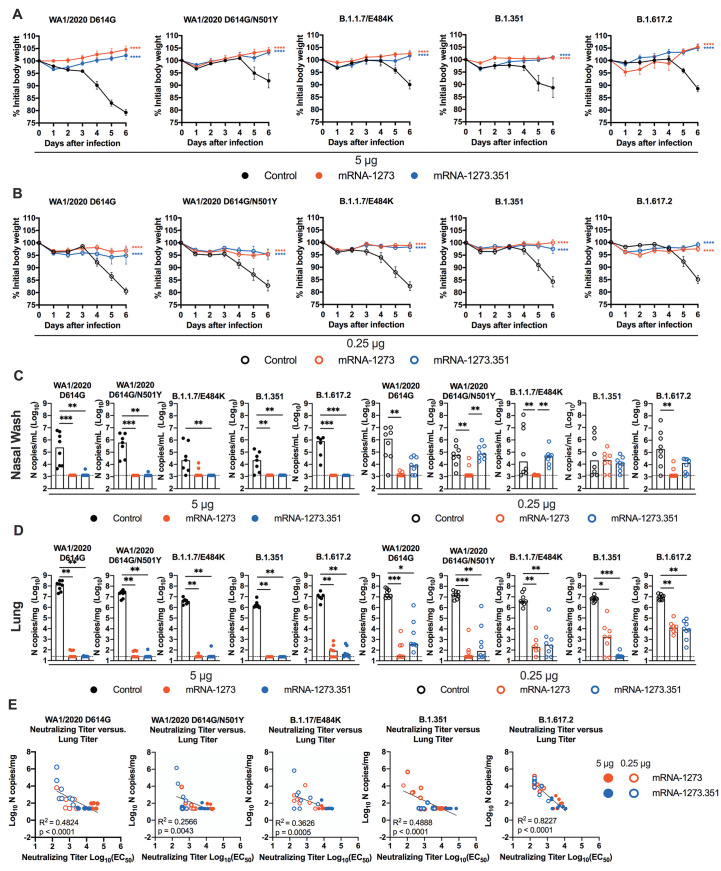
mRNA vaccination confers protection against SARS-CoV-2 infection in K18-hACE2 transgenic mice. Seven-week-old female K18-hACE2 mice were immunized and boosted with 5 or 0.25 μg of mRNA vaccines as described in Fig. 4A. Four weeks after boosting, mice were challenged with 10^3^ to 3 × 10^4^ FFU of WA1/2020 D614G, WA1/2020 N501Y/D614G, B.1.1.7/E484K, B.1.351, or B.1.617.2, depending on the strain. (**A** and **B**) Body weight change was measured over time. Data are presented as mean ± SEM (n = 8, two experiments). Data were analyzed by a one-way ANOVA of area under the curve from 2 to 4 dpi with Dunnett’s post-test, comparison to control immunized group: ****P < 0.0001. (**C** and **D**) Viral burden at 6 dpi in the nasal washes (C) and lungs (D) was assessed by qRT-PCR of the *N* gene after challenge of immunized mice (n = 6 to 8 mice per group, two experiments). Boxes illustrate median values, and dotted line shows LOD. Data were analyzed by a one-way Kruskal-Wallis ANOVA with Dunn’s post-test, comparison among all immunization groups: *P < 0.05; **P < 0.01; ***P < 0.001). (**E**) Correlation analyses are shown comparing serum neutralizing antibody concentrations three weeks after boosting plotted against lung viral titer (6 dpi) in K18-hACE2 mice after challenge with the indicated SARS-CoV-2 strain. Pearson’s correlation P and R2 values are indicated as insets. Closed symbols 5 μg vaccine dose; open symbols, 0.25 μg vaccine dose.

**High dose mRNA vaccines confer protection in K18-hACE2 transgenic mice.** We next evaluated the protective activity of the mRNA vaccines in K18-hACE2 mice. Three to four weeks after boosting, mice were challenged by the intranasal route with WA1/2020 D614G, WA1/2020 N501Y/D614G, B.1.1.7/E484K, B.1.351, or B.1.617.2 strains. Compared to the control mRNA vaccine, the 5 μg and 0.25 μg doses of mRNA-1273 and mRNA-1273.351 vaccines all prevented the weight loss occurring between 3 and 6 dpi (**Fig. 5A and B**).

At 6 dpi, mice were euthanized, and nasal washes, lungs, and brains were collected for viral burden analysis (**Fig. 5C and D****, and fig. S4A**). In the nasal washes of control mRNA-vaccinated K18-hACE2 mice, moderate amounts (about 10^5^ copies of *N* per mL) of viral RNA were measured after challenge with WA1/2020 D614G, WA1/2020 N501Y/D614G, B.1.1.7/E484K, B.1.351, or B.1.617.2 strains, although some variability was observed (**Fig. 5C**). In comparison, in the lungs of control mRNA-vaccinated K18-hACE2 mice, higher and more uniform amounts (about 10^7^ copies of *N* per mg) of viral RNA were detected after challenge with all SARS-CoV-2 strains (**Fig. 5D**). The viral burden in the brains of control RNA-vaccinated K18-hACE2 mice showed some variability, as seen previously (*22*), with many but not all animals showing substantial infection (10^8^ copies of *N* per mL) (**fig. S4A**). The high 5 μg dose of mRNA-1273 or mRNA-1273.351 vaccines protected against infection in nasal washes, lung, and brain, with no viral breakthrough regardless of the challenge strain. After the 0.25 μg dose immunization with mRNA-1273, a loss of protection against infection in the nasal washes and lungs (6 of 8 mice) was observed after challenge with B.1.351 and in the lungs only after challenge with B.1.1.7/E484K (6 of 7 mice) or B.1.617.2 (8 of 8 mice) viruses. After the 0.25 μg dose immunization with mRNA-1273.351, incomplete protection against infection in the nasal washes, lungs, and brain also was observed after challenge with WA1/2020 D614G (6, 7, and 4 of 8 mice, respectively), WA1/2020 D614G/N501Y (8, 4, and 6 of 8 mice, respectively), B.1.1.7/E484K (8, 6, and 3 of 8 mice, respectively), and B.1.617.2 (7, 8, and 5 of 8 mice, respectively). The 0.25 μg dose of the mRNA-1273.351 vaccine protected better against lung and brain infection by the homologous B.1.351 virus than against other strains.

We explored whether vaccine-induced neutralizing antibody titers correlated with protection after challenge with WA1/2020 D614G, WA1/2020 N501Y/D614G, B.1.1.7/E484K, B.1.351, or B.1.617.2 viruses. In general, serum neutralizing antibody titers exhibited an inverse correlation with amounts of viral RNA in the lung (**Fig. 5E**) for all viruses, with more infection occurring in animals with lower neutralization titers. However, for WA1/2020 D614G, WA1/2020 N501Y/D614G, B.1.1.7/E484K, and B.1.351, some of the animals with low neutralization titers were still protected against infection in the lung. The correlation was most linear for B.1.617.2-challenged animals, with a minimum neutralizing titer of approximately 2,000 required to completely prevent infection at 6 dpi. Most of the breakthrough B.1.617.2 infections occurred with the 0.25 μg dose of mRNA vaccines. The threshold for complete protection in the lung after virus challenge varied somewhat with lower amounts required for WA1/2020 D614G and WA1/2020 N501Y/D614G. When we compared body weight change in K18-hACE2 mice at 6 dpi with neutralizing titers, a linear relationship was observed with all challenge viruses except B.1.351 (**fig. S4B**). The best correlation was seen after B.1.617.2 challenge, with greater weight loss in mice immunized with the 0.25 μg vaccine dose and having lower serum neutralizing antibody titers.

Because a pro-inflammatory host response to SARS-CoV-2 infection can contribute to pulmonary pathology and severe COVID-19, we assessed the ability of the mRNA vaccines to suppress cytokine and chemokine responses in the lung after virus challenge (**fig. S5**). For these studies, K18-hACE2 mice were immunized and boosted with 5 or 0.25 μg of control, mRNA-1273 or mRNA-1273.351 vaccines and then challenged with WA1/2020 N501Y/D614G, B.1.351, or B.1.617.2. SARS-CoV-2 infection of control mRNA vaccinated K18-hACE2 mice resulted in high expression of several pro-inflammatory cytokines and chemokines in lung homogenates, including granulocyte colony-stimulating factor (G-CSF), IFN-γ, interleukin (IL)-1β, IL-6, CXCL1, CXCL5, CXCL9, CXCL10, CCL2, and CCL4. Pro-inflammatory cytokine and chemokines in the lung at 6 dpi generally were decreased in all mice vaccinated with 5 μg doses of mRNA-1273 or mRNA-1273.351, regardless of the challenge virus (**fig. S5A and B**). Although this pattern was also observed for the 0.25 μg dose of both mRNA vaccines, some cytokines and chemokines (such as IL-1 β, IL-6, CXCL9, and CXCL10) remained elevated, especially after challenge with B.1.617.2 (**fig. S5C and D**).

**Fig. 6.**
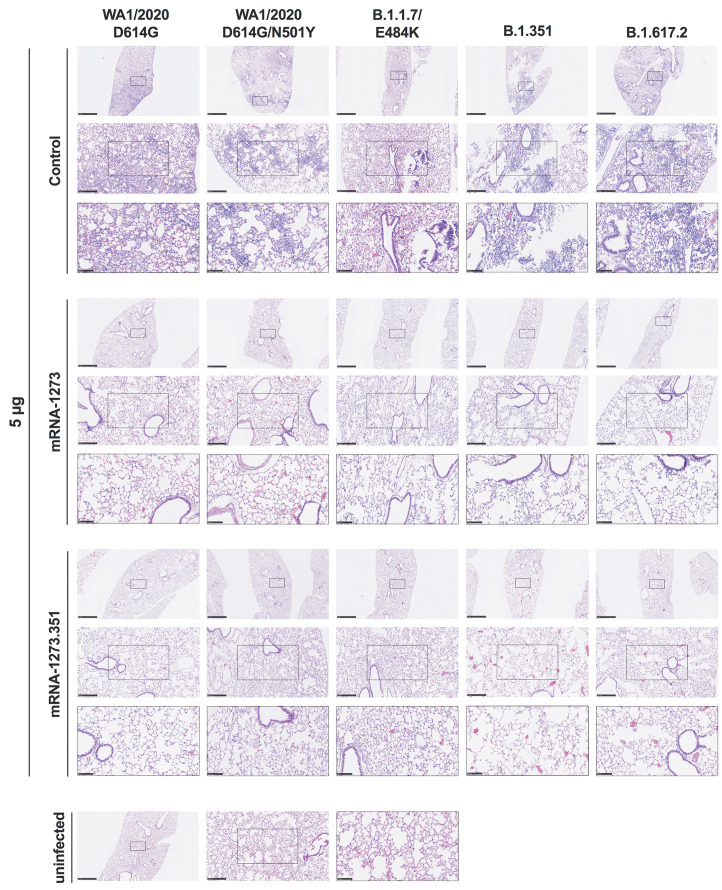
**High-dose mRNA vaccine protects against lung pathology in K18-hACE2 transgenic mice**. Seven to nine-week-old female K18-hACE2 transgenic mice were immunized, boosted with 5 μg of mRNA vaccines, and challenged with WA1/2020, WA1/2020 N501Y/D614G, B.1.1.7/E484K, B.1.351, or B.1.617.2. as described in Fig. 5. Hematoxylin and eosin staining of lung sections harvested at 6 dpi or from an uninfected animal are shown. Images show low- (top; scale bars, 1 mm), medium- (middle; scale bars, 250 μm), and high-power (bottom; scale bars, 100 μm) magnification for each sample. Representative images of multiple lung sections are presented from n = 2 per group.

**Fig. 7.**
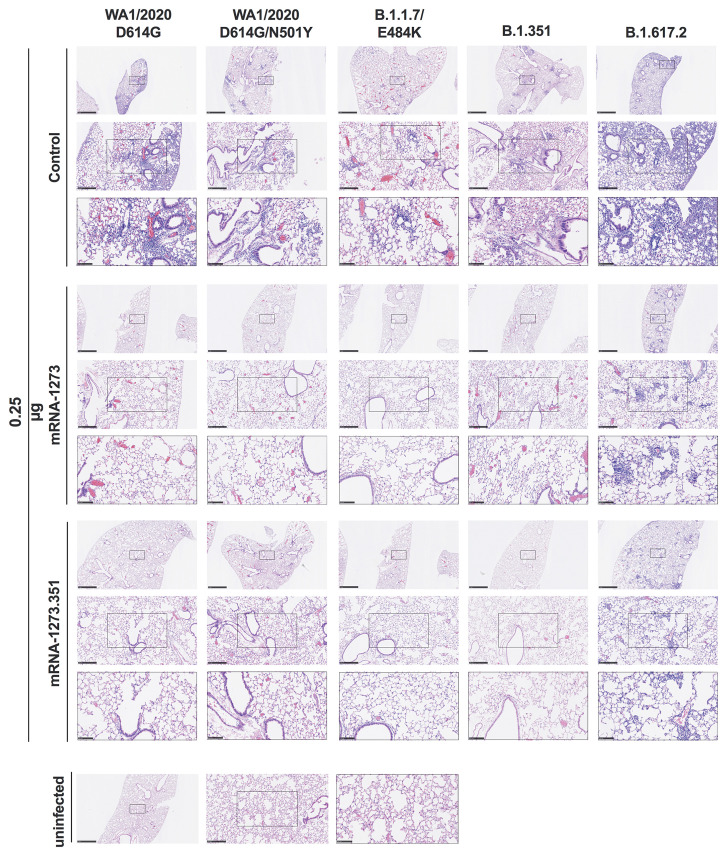
Low-dose mRNA vaccine confers protection against lung pathology against most tested strains of SARS-CoV-2 in K18-hACE2 transgenic mice. Seven to nine-week-old female K18-hACE2 transgenic mice were immunized, boosted with 0.25 μg of mRNA vaccines, and challenged with WA1/2020, WA1/2020 N501Y/D614G, B.1.1.7/E484K, B.1.351, or B.1.617.2. as described in Fig. 5. Hematoxylin and eosin staining of lung sections harvested at 6 dpi or from an uninfected animal are shown. Images show low- (top; scale bars, 1 mm), medium- (middle; scale bars, 250 μm), and high-power (bottom; scale bars, 100 μm). Representative images of multiple lung sections are presented from n = 2 per group.

We evaluated the ability of the mRNA-1273 and mRNA-1273.351 vaccines to prevent disease in K18-hACE2 mice by performing histological analysis of lung tissues from immunized animals that were challenged with WA1/2020 D614G, WA1/2020 N501Y/D614G, B.1.1.7/E484K, B.1.351, or B.1.617.2. As expected, lung sections obtained at 6 dpi from mice immunized with the control mRNA vaccine and challenged with any of the SARS-CoV-2 strains showed severe pneumonia characterized by immune cell infiltration, alveolar space consolidation, vascular congestion, and interstitial edema (**Fig. 6**** and ****7**). In comparison, mice immunized with the 5 μg dose of mRNA-1273 or mRNA-1273.351 did not develop lung pathology, with histological findings similar to uninfected K18-hACE2 mice (**Fig. 6**). Mice immunized with the 0.25 μg dose of the mRNA vaccines however, showed different results (**Fig. 7**). Mice vaccinated with mRNA-1273 showed few, if any, pathological changes after WA1/2020 D614G, WA1/2020 N501Y/D614G, or B.1.1.7/E484K challenge. Nonetheless, some mRNA-1273 vaccinated mice challenged with B.1.351 showed pulmonary vascular congestion and mild lung inflammation. Mice vaccinated with mRNA-1273.351 showed almost complete protection after WA1/2020 D614G, B.1.1.7/E484K, or B.1.351 challenge, whereas scattered inflammation and alveolar septal thickening was apparent in sections from some WA1/2020 N501Y/D614G challenged mice. Of note, lung sections from mice vaccinated with the lower 0.25 μg dose mRNA-1273 or mRNA-1273.351 and challenged with B.1.617.2 showed evidence of viral pneumonia with prominent foci of immune cells inflammation and airspace consolidation. Thus, low doses of the original mRNA-1273 or the variant mRNA vaccines do not fully protect K18-hACE2 mice from challenge with B.1.617.2 and result in mild to moderate infection and lung pathology.

## DISCUSSION

Robust vaccine-induced immune responses and sustained protective activity against emerging SARS-CoV-2 variants are needed to limit human disease and curtail the COVID-19 pandemic. A concern in the field is whether immunity generated by vaccines will lose activity against VOC with mutations or deletions in regions of the spike protein recognized by neutralizing antibodies. In the current study, we evaluated the immunogenicity and protective activity of high- and low-dose formulations of Moderna mRNA vaccines targeting historical (mRNA-1273) or variant (mRNA-1273.351) strains. The low-dose vaccine study arm was designed to model individuals with suboptimal immune responses and assess for possible strain-specific breakthrough infections.

Immunization of 129S2 or K18-hACE2 transgenic mice with mRNA-1273, mRNA-1273.351, or the mRNA-1273.211 mixture induced neutralizing antibodies against spike protein in serum against historical WA1/2020 and several key VOC. Challenge studies performed approximately one month after the second vaccine dose showed robust protection against weight loss and lung pathology with all high-dose vaccines and infecting SARS-CoV-2 strains. Nonetheless, the low-dose vaccine formulations showed evidence of viral infection breakthrough and lung pathological changes consistent with pneumonia, especially with the B.1.617.2 strain, which correlated with lower strain-specific neutralizing antibody titers in mice. In general, variant-specific vaccine designs appeared to induce greater antibody responses and confer more protection against homologous virus strains.

Our experiments expand upon a preliminary immunogenicity study, which showed that vaccination of H-2^d^ BALB/c mice with mRNA-1273.351 resulted in high serum neutralizing antibody titers against the B.1.351 lineage, whereas the mRNA-1273.211 vaccine induced broad cross-variant neutralization (*19*). We performed experiments with two H-2^b^-expressing strains, 129S2 and K18-hACE2 C57BL/6, and observed some similarities and differences. In K18-hACE2 mice, the mRNA-1273 vaccine, which encodes for the Wuhan-1 pre-fusion stabilized spike, induced higher neutralizing titers against WA1/2020 strains but lower responses against viruses containing E484K mutations in spike (B.1.1.7/E484K and B.1.351), which agrees with recent immunization studies in NHPs (*14*). Reciprocally, the mRNA-1273.351 vaccine, which encodes for the B.1.351 pre-fusion stabilized spike, induced higher neutralizing titers against B.1.1.7/E484K and B.1.351. In 129S2 mice, only the mRNA-1273.351 vaccine induced a lower neutralizing response against WA1/2020 D614G, as the remainder of the neutralizing antibody responses were largely equivalent between vaccines. However, in both K18-hACE2 and 129S2 mice, the mRNA-1273 and mRNA-1273.351 vaccines induced antibody responses that neutralized B.1.617.2 less efficiently than the other SARS-CoV-2 strains. Analysis of serum antibodies and B cell repertoires against SARS-CoV-2 VOC from ongoing human clinical trials comparing mRNA-1273 and mRNA-1273.351 vaccines will be needed to corroborate our results obtained in small animal models. Indeed, the differences in neutralizing antibody titers induced by mRNA-1273 against WA1/2020 D614G and B.1.351 in mice were smaller in magnitude than that seen in humans one month after boosting but were more similar to that observed six months after boosting (*23*).

The greatest loss in antibody neutralization (both 129S2 and K18-hACE2 mice) and protection (K18-hACE2 mice) consistently occurred with the B.1.617.2 variant. These results contrast with a recent longitudinal study in humans immunized with mRNA-1273, which showed lower neutralizing titers of serum antibody against B.1.351 than other VOC, including B.1.617.2, although those data were collected using pseudovirus rather than live virus neutralization assays (*23*). Another study using a live virus neutralization assay and human serum obtained three weeks post immunization with the BNT162b2 mRNA vaccine also showed lower neutralizing titers against B.1.351 than B.1617.2, though this trend reversed at 6 months (*24*). In our experiments with live virus, the loss of neutralizing activity was equivalent if not greater for B.1.617.2 than B.1.351, as reported by others with human serum samples from individuals infected with B.1.351 or P.1 (*25*). Based on sequence changes in the spike protein (B.1.617.2: T19R, 156del, 157del, R158G, L452R, T478K, D614G, P681R, and D950N; and B.1.351: D80A, D215G, 241del, 242del, 243del, K417N, E484K, N501Y, D614G, A701V) and known binding sites in the receptor binding motif of neutralizing antibodies (at residue E484), it is not apparent why neutralizing activity and protection in mice were lower against B.1.617.2 than B.1.351, although there was an inverse correlation with titers of neutralizing antibody and B.1.617.2 burden in the lung. Nonetheless, mutations in the B.1.617.2 alter key antigenic sites and can abrogate recognition by neutralizing antibodies (*26*). Other possible explanations for the loss of potency of antibodies against B.1.617.2 include differential display of B.1.617.2 spike proteins on the surface of infected cells and engagement of Fc effector functions (*27*, *28*) or differential ability of antibodies to block cell-to-cell spread in a strain-dependent manner (*29*). Our observation of B.1.617.2 infection and lung disease in low-dose mRNA-vaccinated K18-hACE2 mice corresponds to descriptions of B.1.617.2 breakthrough infections in vaccinated humans, some of which have required hospitalization (*30*, *31*).

We note several limitations in our study. First, the studies in 129S2 mice precluded challenge with B.1.617.2, as it does not infect mice because it lacks an N501Y mutation. The generation of recombinant SARS-CoV-2 strains with spike genes encoding B.1.617.2 and an N501Y mutation could overcome this limitation. Second, female 129S2 and K18-hACE2 mice were used to allow for group caging of the large cohorts required for these multi-arm vaccination studies. Follow-up experiments in male mice are needed to confirm these results are not sex-biased. Third, differences in the repertoire of antibodies in mice and humans could contribute to the relative differences in neutralization potency of serum against B.1.617.2 and B.1.351 viruses. Fourth, we used historical, variant, or mixed mRNA vaccine formulations with homologous boosting schemes. Animals studies that test heterologous boosting (mRNA-1273 prime followed by mRNA-1273.351 boost) (*19*) also are needed to support clinical trials. Fifth, our studies focused on immunogenicity and protection in two strains of mice because of the ability to set up large animal cohorts and the tools available for analysis. These results require confirmation in other animal models of SARS-CoV-2 infection including hamsters and non-human primates (*32*). Sixth, we did not establish immunological correlates of vaccine protection or failure for all vaccine and challenge strain pairs. Although some relationships were more predictive (low B.1.617.2 neutralizing titers and viral burden in the lung), others were not.

Our studies in 129S2 and K18-hACE2 mice with parental and modified mRNA vaccines show robust immunogenicity and protection against multiple SARS-CoV-2 strains when high-dose immunization schemes are used, although some differences in immunity are seen, particular with vaccines against selected variants. Although the lower dose of mRNA vaccines generally protected against matched virus challenge infection (mRNA-1273 vaccination and WA1/2020 challenge or mRNA-1273.351 vaccination and B.1.351 challenge), breakthrough events were seen with some non-matched challenges (mRNA-1273 vaccination and B.1.351 challenge or mRNA-1273.351 vaccination and WA1/2020 challenge). As the low dose of mRNA-1273 and 1273.351 vaccines induced lower neutralizing titers and protected less against challenge with the B.1.617.2 variant, higher titers will be needed to minimize B.1.617.2 infection, transmission, and disease. Although studies in humans are required, boosting with historical or variant vaccines might be required to prevent breakthrough events for individuals with suboptimal responses. Indeed, booster doses for vaccines recently were approved for some populations in the United States and other parts of the world based on recent breakthrough infection data (*33*).

## MATERIALS AND METHODS

**Study Design.** The goal of this study was to evaluate the immunogenicity and efficacy of a high or low-dose of mRNA vaccines (mRNA-1273, mRNA-1273.351, or mRNA-1273.211) against emerging SARS-CoV-2 strains using two different mouse models (129S2 and K18-hACE2). Mice were immunized, boosted three weeks later, and immune responses were analyzed. Approximately three weeks later, animals were challenged with different SARS-CoV-2 strains, and clinical, virological, immunological, and pathological outcomes were measured. All data collected was included without exclusion of outliers. Mice were randomly assigned to cages, and investigators performing the immunological analyses were blinded. Sample sizes were chosen based on power analysis estimates and prior experience in evaluating differences in serological responses and viral infection in mice. All experiments in mice were repeated on two separate occasions.

**Cells.** African green monkey Vero-TMPRSS2 (*34*) and Vero-hACE2-TMPRRS2 (*7*) cells were cultured at 37°C in Dulbecco’s Modified Eagle medium (DMEM) (Thermo Fisher Scientific) supplemented with 10% fetal bovine serum (FBS), 10 mM HEPES pH 7.3, 1 mM sodium pyruvate, 1× non-essential amino acids, and 100 U/mL of penicillin–streptomycin (Thermo Fisher Scientific). Vero-TMPRSS2 cells were supplemented with 5 μg/mL of blasticidin. Vero-hACE2-TMPRSS2 cells were supplemented with 10 μg/mL of puromycin. All cells routinely tested negative for mycoplasma using a polymerase-chain reaction (PCR)-based assay.

**Viruses**. The WA1/2020 recombinant strain with substitutions (D614G or N501Y/D614G) were obtained from an infectious cDNA clone as described previously (*35*). The B.1.351, B.1.1.7/E484K, and B.1.617.2 strains were obtained from nasopharyngeal isolates. All viruses were passaged once in Vero-TMPRSS2 cells and subjected to next-generation sequencing (*7*) to confirm the introduction and stability of substitutions. All virus experiments were performed in an approved biosafety level 3 (BSL-3) facility.

**Mice.** Animal studies were carried out in accordance with the recommendations in the Guide for the Care and Use of Laboratory Animals of the National Institutes of Health. The protocols were approved by the Institutional Animal Care and Use Committee at the Washington University School of Medicine (assurance number A3381–01). Virus inoculations were performed under anesthesia that was induced and maintained with ketamine hydrochloride and xylazine, and all efforts were made to minimize animal suffering. Heterozygous K18-hACE2 C57BL/6J mice (strain: 2B6.Cg-Tg(K18-ACE2)2Prlmn/J, Cat # 34860) and 129S2 mice (strain: 129S2/SvPasCrl, Cat # 287) were obtained from The Jackson Laboratory and Charles River Laboratories, respectively. Animals were housed in groups and fed standard chow diets.

**Pre-clinical vaccine mRNA and lipid nanoparticle production process.** A sequence-optimized mRNA encoding prefusion-stabilized Wuhan-Hu-1 (mRNA-1273) or B.1.351-variant (mRNA-1273.351) SARS-CoV-2 S-2P protein was synthesized in vitro using an optimized T7 RNA polymerase-mediated transcription reaction with complete replacement of uridine by N1m-pseudouridine (*36*). The reaction included a DNA template containing the immunogen open-reading frame flanked by 5′ untranslated region (UTR) and 3′ UTR sequences, and was terminated by an encoded polyA tail. After transcription, the cap-1 structure was added using the vaccinia virus capping enzyme (New England Biolabs). The mRNA was purified by oligo-dT affinity purification, buffer exchanged by tangential flow filtration into sodium acetate, pH 5.0, sterile filtered, and kept frozen at –20°C until further use.

The mRNA was encapsulated in a lipid nanoparticle through a modified ethanol-drop nanoprecipitation process described previously (*37*). Ionizable, structural, helper, and polyethylene glycol lipids were briefly mixed with mRNA in an acetate buffer, pH 5.0, at a ratio of 2.5:1 (lipid:mRNA). The mixture was neutralized with Tris-HCl, pH 7.5, sucrose was added as a cryoprotectant, and the final solution was sterile-filtered. Vials were filled with formulated lipid nanoparticle and stored frozen at –20°C until further use. The vaccine product underwent analytical characterization, which included the determination of particle size and polydispersity, encapsulation, mRNA purity, double-stranded RNA content, osmolality, pH, endotoxin, and bioburden, and the material was deemed acceptable for in vivo study.

**Antigens.** Recombinant soluble spike proteins from different SARS-CoV-2 strains were expressed as described (*20*, *38*). Briefly, mammalian cell codon-optimized nucleotide sequences coding for the soluble ectodomain of the spike protein of SARS-CoV-2 including a C-terminal thrombin cleavage site, T4 foldon trimerization domain, and hexahistidine tag were cloned into mammalian expression vector pCAGGS. The spike protein sequence was modified to remove the polybasic cleavage site (RRAR to A), and two pre-fusion stabilizing proline mutations were introduced (K986P and V987P, wild type Wuhan-Hu-1 numbering). Recombinant proteins were produced in Expi293F cells (Thermo Fisher Scientific) by transfection of DNA using the ExpiFectamine 293 Transfection Kit (Thermo Fisher Scientific). Supernatants were harvested 3 days post-transfection, and recombinant proteins were purified using Ni-NTA agarose (Thermo Fisher Scientific), then buffer exchanged into phosphate-buffered saline (PBS) and concentrated using Amicon Ultracel centrifugal filters (EMD Millipore).

**ELISA.** Assays were performed in 96-well microtiter plates (Thermo Fisher Scientific) coated with 50 μL of recombinant spike protein from Wuhan-1 SARS-CoV-2 or variant viruses B.1.1.7, B.1.351, or B.1.617.2. Plates were incubated at 4°C overnight and then blocked with 200 μL of 3% non-fat dry milk (AmericanBio) in PBS containing 0.1% Tween-20 (PBST) for 1 hour at room temperature. Serum samples were serially diluted in 1% non-fat dry milk in PBST and added to the plates. Plates were incubated for 2 hours at room temperature and then washed 3 times with PBST. Goat anti-mouse IgG-horseradish peroxidase (HRP, Sigma-Aldrich, 1:9000) was diluted in 1% non-fat dry milk in PBST before adding to the wells and incubating for 1 hour at room temperature. Plates were washed 3 times with PBST before the addition of peroxidase substrate (SigmaFAST o-phenylenediamine dihydrochloride, Sigma-Aldrich). Reactions were stopped by the addition of 3 M hydrochloric acid. Optical density (OD) measurements were taken at 490 nm, and endpoint titers were calculated in excel using a 0.15 OD 490 nm cutoff. Graphs were generated using GraphPad Prism v9.

**Focus reduction neutralization test.** Serial dilutions of serum samples were incubated with 10^2^ focus-forming units (FFU) of different strains of SARS-CoV-2 for 1 hour at 37°C. Antibody-virus complexes were added to Vero-TMPRSS2 cell monolayers in 96-well plates and incubated at 37°C for 1 hour. Subsequently, cells were overlaid with 1% (w/v) methylcellulose in Eagle’s Minimal Essential medium (Thermo Fisher Scientific). Plates were harvested 30 hours later by removing overlays and fixed with 4% paraformaldehyde (PFA) in PBS for 20 min at room temperature. Plates were washed and sequentially incubated with an oligoclonal pool of SARS2-2, SARS2-11, SARS2-16, SARS2-31, SARS2-38, SARS2-57, and SARS2-71 (*39*) anti-spike protein antibodies and HRP-conjugated goat anti-mouse IgG (Sigma Cat # A8924, RRID: AB_258426) in PBS supplemented with 0.1% saponin and 0.1% bovine serum albumin. SARS-CoV-2-infected cell foci were visualized using TrueBlue peroxidase substrate (KPL) and quantitated on an ImmunoSpot microanalyzer (Cellular Technologies).

**Mouse experiments**. Seven to nine-week-old female 129S2 and K18-hACE2 C57BL/6 mice were immunized and boosted three weeks apart with 5 or 0.25 μg (total dose) of mRNA vaccines (control, mRNA-1273, mRNA-1273.351, or mRNA-1273.211) in 50 μl of PBS by intramuscular injection in the hind leg. The mRNA 1273.211 combination vaccine was mixed from the mRNA-1273 and mRNA-1273.351 vaccines at a 1:1 ratio immediately before usage. Animals were bled at specified time points to obtain serum for immunogenicity analysis. Three to four weeks after boosting, mice were challenged with 10^5^ FFU (129S2) or 10^3^ to 3 × 10^4^ FFU (K18-hACE2) of WA1/2020 D614G (10^4^), WA1/2020 N501Y/D614G (10^3^), B.1.1.7/E484K (10^3^), B.1.351 (10^3^), or B.1.617.2 (3 × 10^4^) of SARS-CoV-2 strains by the intranasal route. Different doses of viruses were used in K18-hACE2 mice to match weight loss and infection data. This approach was necessary as some viruses (WA1/2020 N501Y/D614G, B.1.1.7/E484K, and B.1.351) encode N501Y mutations that enhance pathogenicity in mice (*15*, *16*, *40*). Animals were euthanized at 4 or 6 dpi, and tissues were harvested for virological, immunological, and pathological analyses.

**Measurement of viral burden.** Tissues were weighed and then homogenized with zirconia beads in a MagNA Lyser instrument (Roche Life Science) in 1 ml of DMEM medium supplemented with 2% heat-inactivated FBS. Tissue homogenates were clarified by centrifugation at 10,000 rpm for 5 min and stored at −80°C. RNA was extracted using the MagMax mirVana Total RNA isolation kit (Thermo Fisher Scientific) on the Kingfisher Flex extraction robot (Thermo Fisher Scientific). RNA was reverse transcribed and amplified using the TaqMan RNA-to-CT 1-Step Kit (Thermo Fisher Scientific). Reverse transcription was carried out at 48°C for 15 min followed by 2 min at 95°C. Amplification was accomplished over 50 cycles as follows: 95°C for 15 s and 60°C for 1 min. Copies of SARS-CoV-2 *N* gene RNA in samples were determined using a published assay (*41*).

**Cytokine and chemokine protein measurements.** Lung homogenates were incubated with Triton-X-100 (1% final concentration) for 1 hour at room temperature to inactivate SARS-CoV-2. Homogenates were analyzed for cytokines and chemokines by Eve Technologies Corporation using their Mouse Cytokine Array/Chemokine Array 31-Plex (MD31) platform.

**Lung histology.** Lungs of euthanized mice were inflated with 1-2 mL of 10% neutral buffered formalin using a 3-mL syringe and catheter inserted into the trachea. Lungs were then kept in fixative for 7 days. Tissues were embedded in paraffin, and sections were stained with hematoxylin and eosin. Images were captured using the Nanozoomer (Hamamatsu) at the Alafi Neuroimaging Core at Washington University.

**Peptide restimulation and intracellular cytokine staining.** Two weeks after boosting, splenocytes from vaccinated K18-hACE2 mice were stimulated ex vivo with an H-2D^b^-restricted CD8^+^ or CD4^+^ T cell immunodominant peptide (amino acids 262-270 and 62-76 of the spike protein, respectively; gift of K. Valentine and S. Shresta) for 16 hours at 37°C with brefeldin A (BioLegend, 420601) added for the last 4 hours of incubation. Following blocking with FcγR antibody (clone 93; Thermo Fisher Scientific, Cat # 14-0161-85) cells were stained on ice in PBS with 1 μg/ml of CD45 brilliant ultraviolet (BUV) 395 (clone 30-F11; BD Biosciences Cat # 564279, RRID: AB_2651134), CD4 phycoerythrin (PE, clone GK1.5; BD Biosciences Cat # 553730, RRID: AB_395014), CD8 fluorescein isothiocyanate (FITC, clone 53-6.7; BioLegend Cat # 100706, RRID: AB_312745), and Fixable Aqua Dead Cell Stain (Invitrogen, L34966). Stained cells were fixed and permeabilized with the Foxp3/Transcription Factor Staining Buffer Set (eBiosciences, 00-5523). Intracellular staining was performed with anti-IFN-γ Alexa Fluor 647 (clone XMG1.2; BioLegend Cat # 505814, RRID: AB_493314,), and anti-TNF-α brilliant violet (BV) 605 (clone MP6-XT22; BioLegend Cat # 506329, RRID: AB_11123912). Analysis was performed on a BD LSRFortessa X-20 cytometer, using FlowJo X 10.0 software.

**Antigenic cartography**. A target distance from an individual serum to each virus was derived by calculating the difference between the logarithm (log2) reciprocal neutralization titer for that particular virus and the log2 reciprocal maximum titer achieved by that serum (against any virus). Thus, the higher the reciprocal titer, the shorter the target distance. As the log2 of the reciprocal titer was used, a 2-fold change in titer equates to a fixed change in target distance whatever the magnitude of the actual titers. Antigenic cartography (*42*) then was used to optimize the positions of the viruses and serum relative to each other on a map, minimizing the sum-squared error between map distance and target distance. Each virus is therefore positioned by multiple serum samples, and the serum samples themselves also are positioned only by their distances to the viruses. Hence, serum samples with different neutralization profiles to the virus panel are in separate locations on the map but contribute equally to positioning of the viruses.

**Statistical analysis.** All raw, individual level data are shown in Data File S1. Statistical significance was assigned when *P* values were < 0.05 using Prism Version 10 (GraphPad). Tests (one-way Kruskal-Wallis ANOVA with Dunn’s post-test; one or two-way ANOVA with Tukey’s post-test; one-way ANOVA of area under the curve with Dunnett’s post-test), number of animals (n), median values, and statistical comparison groups are indicated in the figure legends.

## Supplementary Materials

### Other Supplementary Material for this manuscript includes the following:

Data file S1

MDAR Reproducibility Checklist
