# Supplementary material for: Protective activity of mRNA vaccines against ancestral and variant SARS-CoV-2 strains
Source: Sci Transl Med. 2021 Nov 30:eabm3302. doi: 10.1126/scitranslmed.abm3302 (PMC8817234; doi:10.1126/scitranslmed.abm3302)
Supplement: Supplementary file 1 — Figs. S1 to S5 [file scitranslmed.abm3302_sm.pdf]

Science Translational Medicine
AAAS

Supplementary Materials for

Protective activity of mRNA vaccines against ancestral and variant SARS-CoV-2 strains

Baoling Ying *et al.*

Corresponding authors: Larissa B. Thackray, lthackray@wustl.edu; Michael S. Diamond, diamond@wusm.wustl.edu

DOI: 10.1126/scitranslmed.abm3302

The PDF file includes:

Fig. S1 to S5

Other Supplementary Material for this manuscript includes the following:

Data file S1
MDAR Reproducibility Checklist

SUPPLEMENTAL FIGURES AND LEGENDS

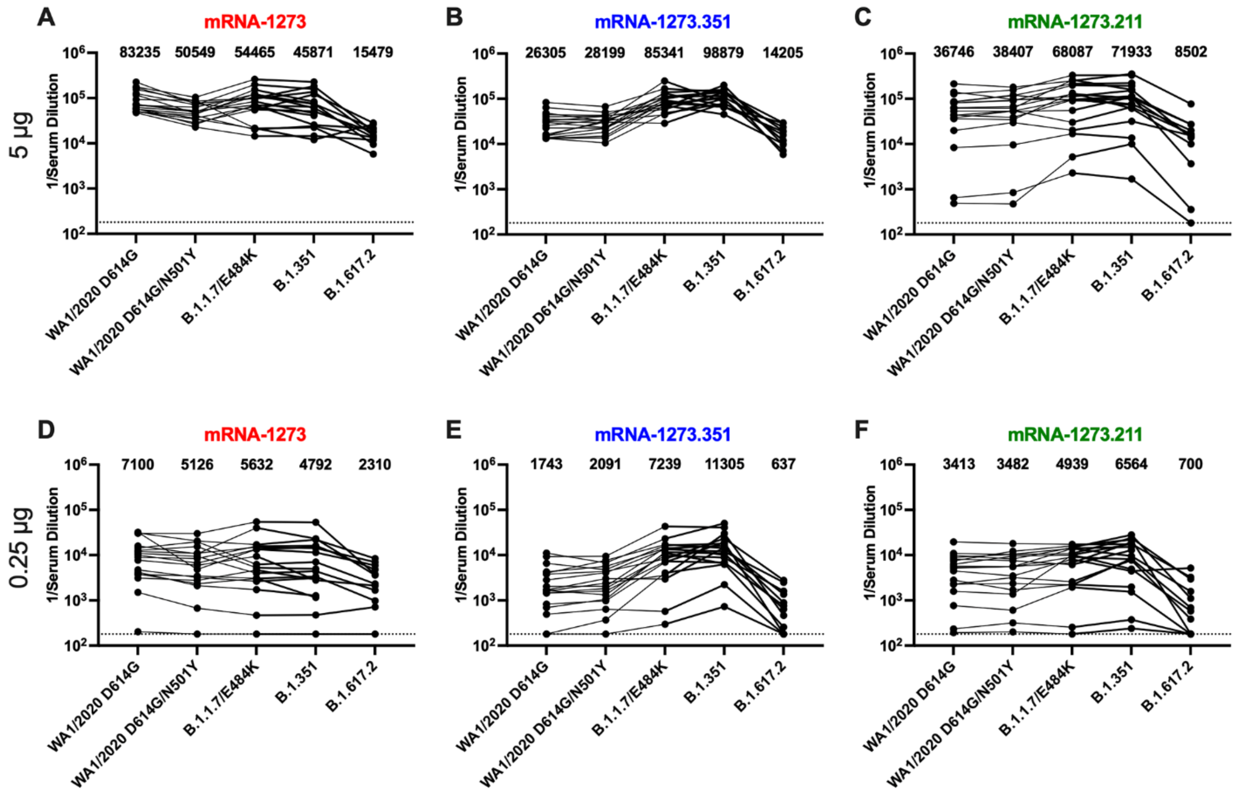

Figure S1. Analysis of serum neutralization of SARS-CoV-2 strains from 129S2 mice immunized with mRNA vaccines. Comparison of neutralizing activity is shown for serum samples against WA1/2020 D614G, WA1/2020 D614G/N501Y, B.1.1.7/E484K, B.1.351, and B.1.617.2. Serum samples were obtained three weeks after boosting with 5 µg (**A to C**) or 0.25 µg (**D to F**) of mRNA vaccines: mRNA-1273 (**A and D**), mRNA-1273.351 (**B and E**), and mRNA-1273.211 (**C and F**). Results are from experiments performed in Fig. 1C to L. Geometric mean neutralization titers (GMT) are shown above each graph, and dotted lines represent the limit of detection (LOD). Solid lines connect data points from the same serum sample across strains.

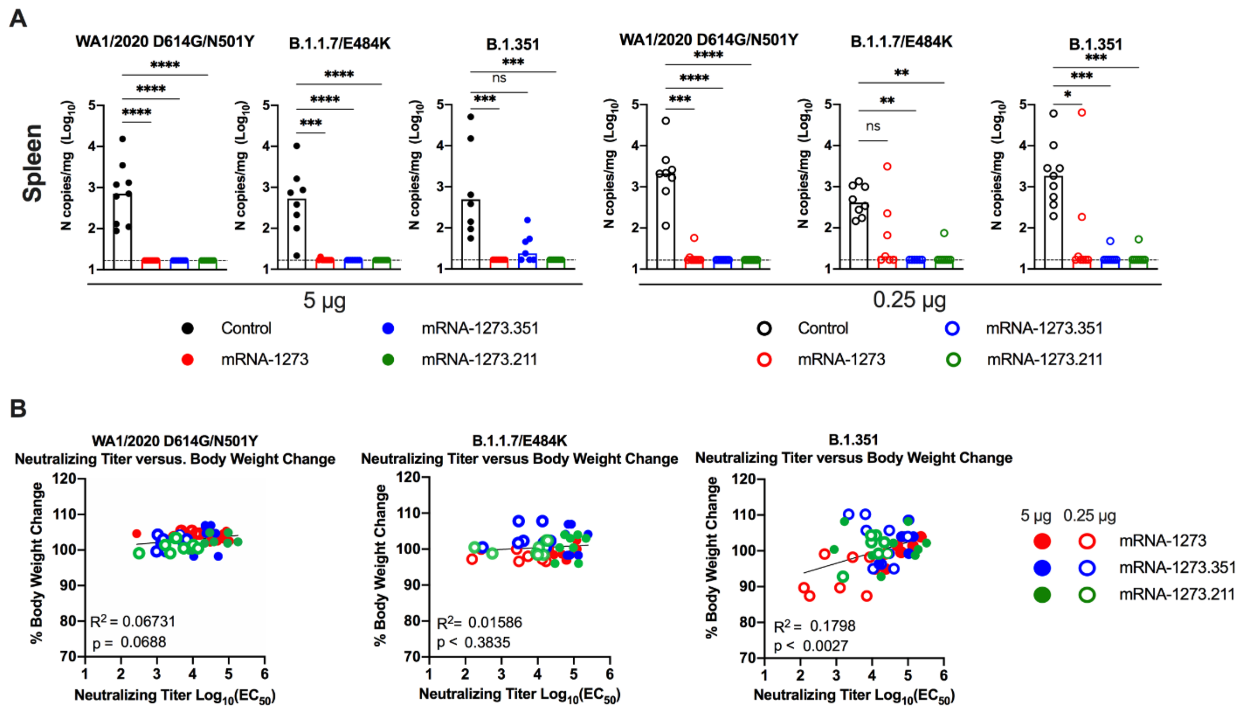

Figure S2. Protection against SARS-CoV-2 infection after mRNA vaccination in 129S2 mice. Seven to nine-week-old female 129S2 mice were immunized and boosted with 5 or 0.25 µg of mRNA vaccines as described in Fig. 1A. Three weeks after boosting, mice were challenged by intranasal inoculation with 10^5 focus-forming units (FFU) of WA1/2020 N501Y/D614G, B.1.1.7/E484K, or B.1.351. (**A**) Viral burden at 4 days post infection (dpi) in the spleen was assessed by qRT-PCR of the *N* gene after challenge of immunized mice with the indicated mRNA vaccines (n = 6 to 8 mice per group, two independent experiments). Boxes illustrate median values and the dotted line shows LOD. Data were analyzed by a one-way Kruskal-Wallis ANOVA with Dunn's post-test comparing among all immunization groups; ns, not significant; ns, $P > 0.05$; $^*P < 0.05$; $^{**}P < 0.01$; $^{***}P < 0.001$; $^{****}P < 0.0001$. (**B**) Correlation analyses are shown comparing serum neutralizing antibody concentrations three weeks after boosting plotted against weight change in 129S2 mice after challenge with the indicated SARS-CoV-2 strain. EC_{50}, half maximal effective concentration. Pearson's correlation *P* and R^2 values are indicated as insets. Closed symbols, 5 µg vaccine dose; open symbols, 0.25 µg vaccine dose.

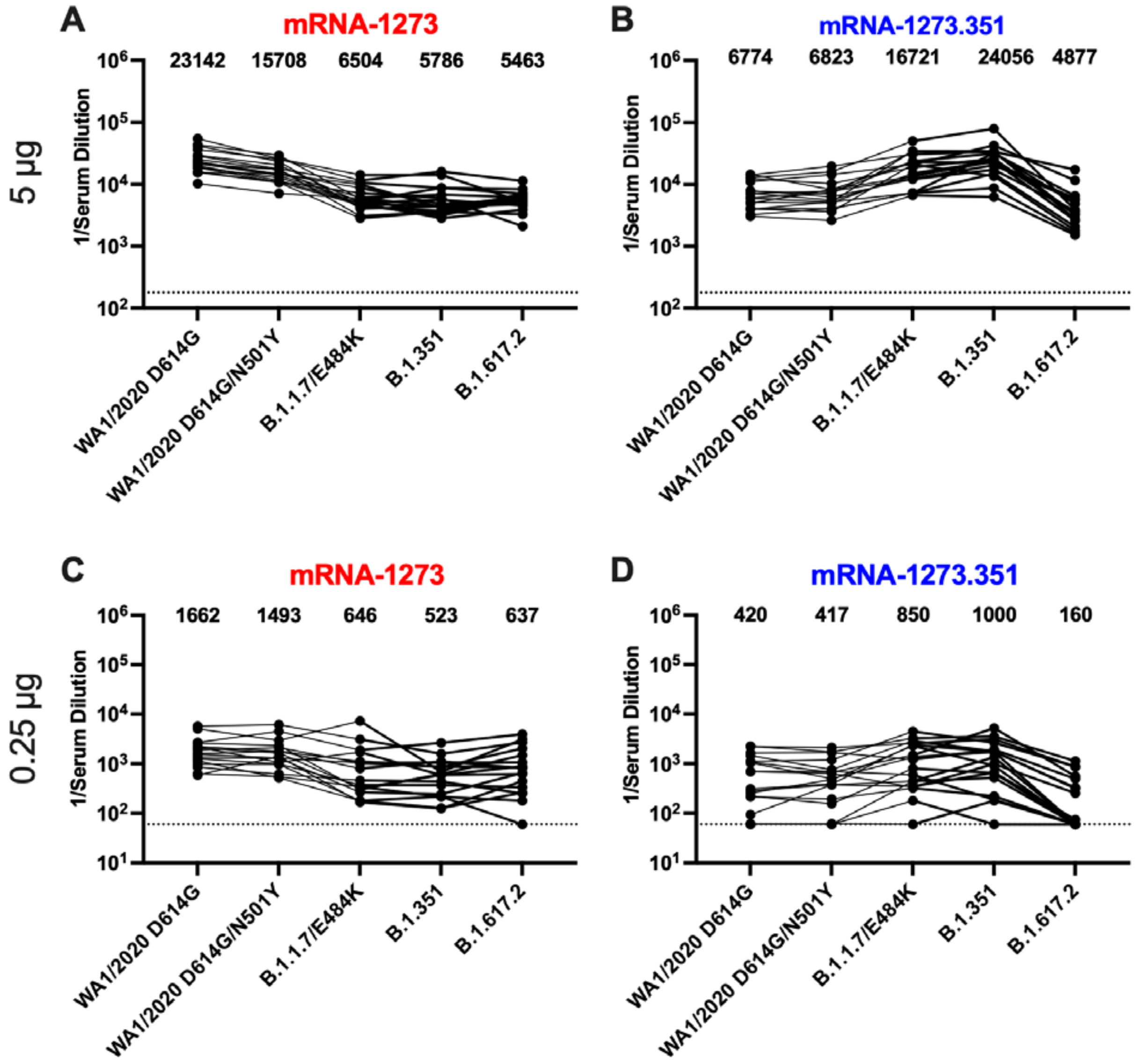

Figure S3. Analysis of serum neutralization of SARS-CoV-2 strains from K18-hACE2 mice immunized with mRNA vaccines. Comparison of neutralizing activity is shown for serum samples against WA1/2020 D614G, WA1/2020 D614G/N501Y, B.1.1.7/E484K, B.1.351, and B.1.617.2. Serum samples were obtained three weeks after boosting with 5 µg (**A and B)** or 0.25 µg (**C and D**) mRNA vaccines: mRNA-1273 (**A and C**) and mRNA-1273.351 (**B and D**). Results are from experiments performed in Fig. 4C to L. GMTs are shown above each graph, dotted line represents the LOD. Solid lines connect data points from the same serum sample across strains.

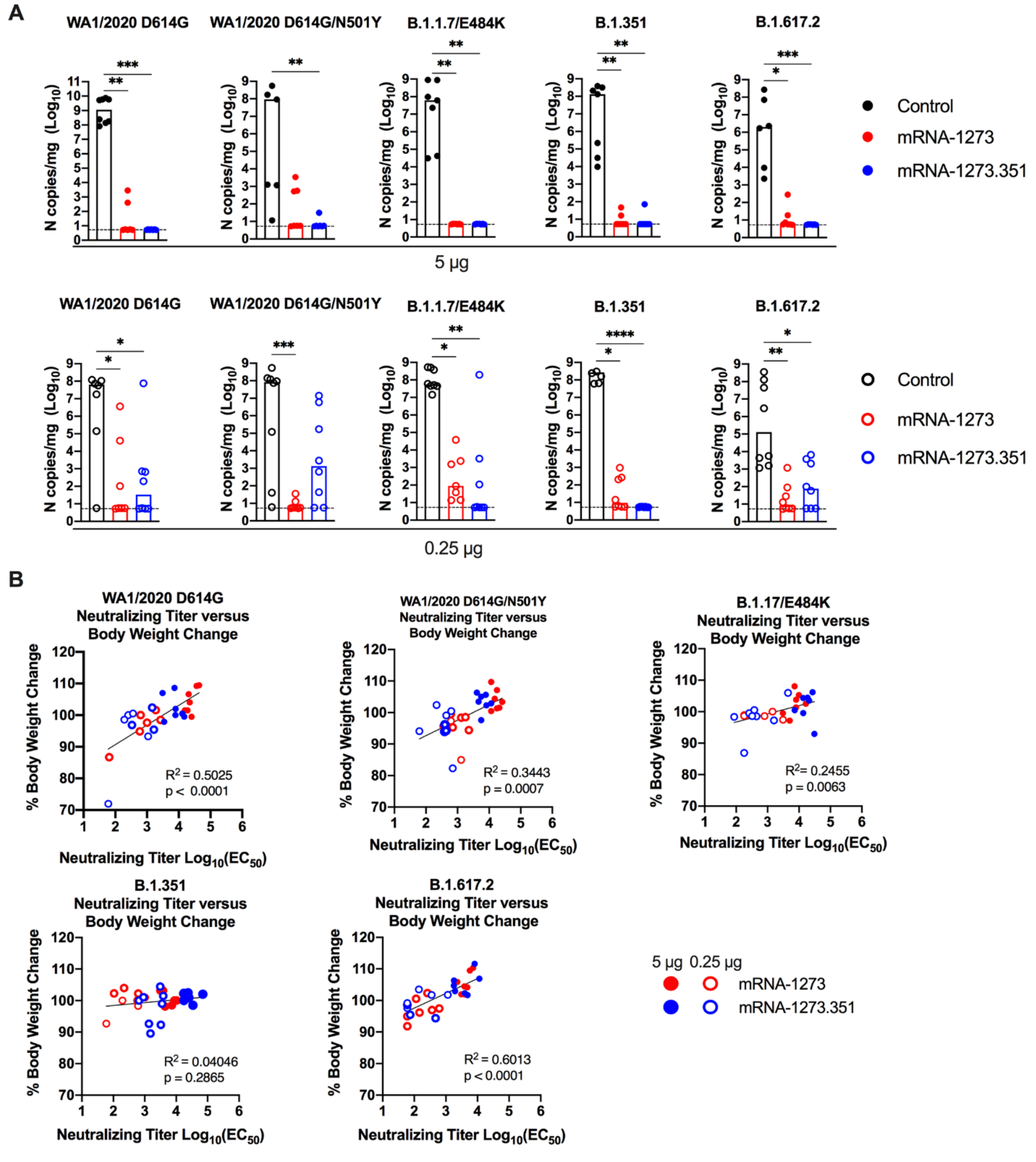

Figure S4. Protection against SARS-CoV-2 infection after mRNA vaccination in K18-hACE2 transgenic mice. Seven-week-old K18-hACE2 mice were immunized and boosted with 5 or 0.25 µg of mRNA vaccines. Controls are shown in black symbols, mRNA-1273 vaccinated mice are shown in red symbols, and mRNA-1273.351 vaccinated mice are shown in blue symbols, as described in Fig. 4A. Three to four weeks after the last boost, mice were challenged by intranasal inoculation with WA1/2020 D614, WA1/2020 N501Y/D614G, B.1.1.7/E484K, B.1.351, or B.1.617.2 as described in Fig. 5. (**A**) Viral burden at 6 dpi in the brain was assessed by qRT-PCR

of the *N* gene after challenge of immunized mice with the indicated mRNA vaccines (n = 6 to 8 mice per group, two independent experiments). Boxes illustrate median values, and dotted lines shows LOD. Data were analyzed using a one-way Kruskal-Wallis ANOVA with Dunn's post-test to compare among all immunization groups; **P* < 0.05; ***P* < 0.01; ****P* < 0.001; *****P* < 0.0001. (**B**) Correlation analyses comparing serum neutralizing antibody concentrations three weeks after boosting plotted against weight change in K18-hACE2 mice after challenge with the indicated SARS-CoV-2 strain are shown. Pearson's correlation *P* and R^2 values are indicated as insets. Closed symbols, 5 µg vaccine dose; open symbols, 0.25 µg vaccine dose.

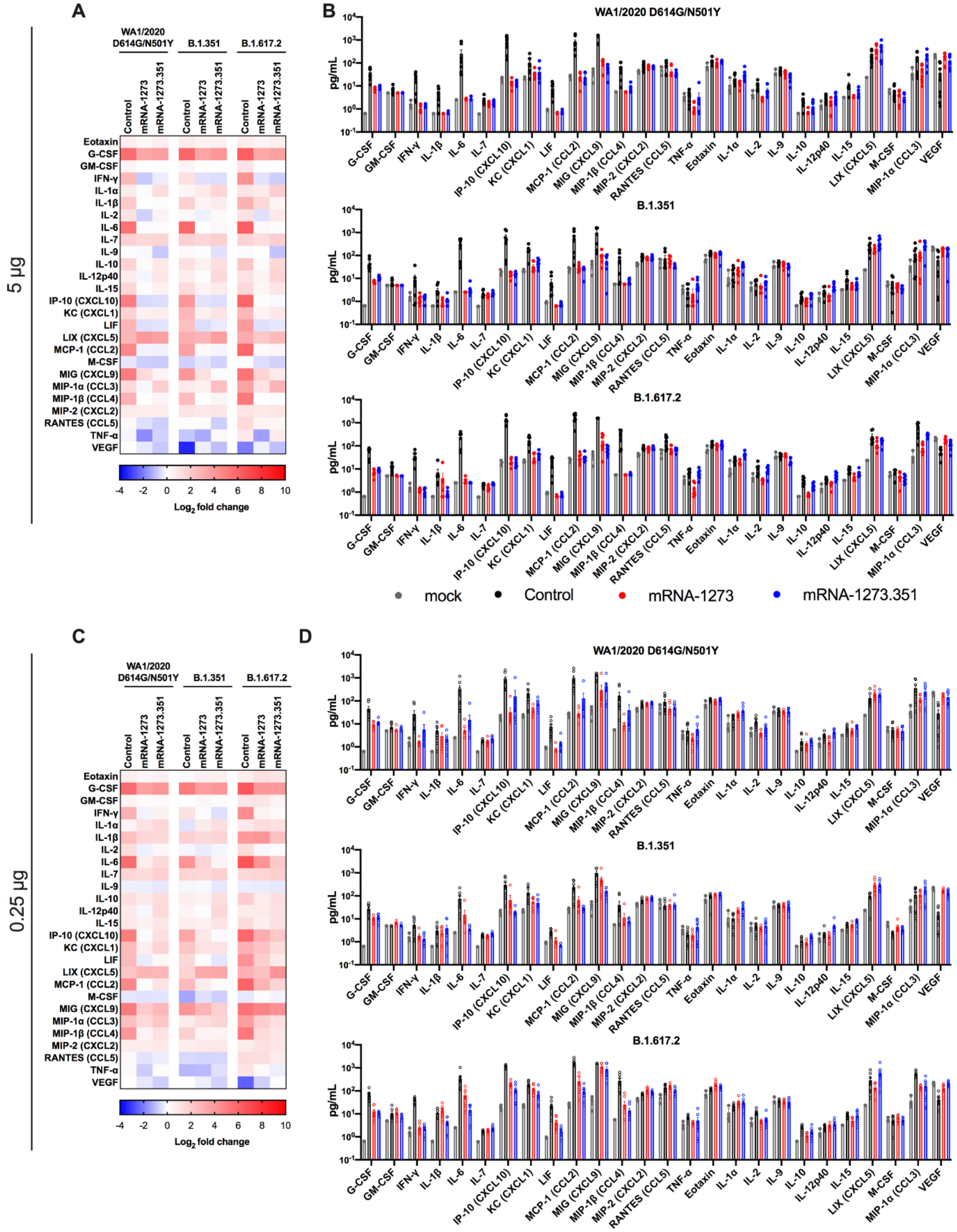

Figure S5. Cytokine induction in lungs after mRNA vaccination and SARS-CoV-2 challenge. Cytokines from mice immunized with 5 µg (**A and B**) or 0.25 µg (**C and D**) dose of mRNA vaccines were measured by multiplex platform in lung tissues isolated from SARS-CoV-2-infected mice at

6 dpi. (**A and C**) For each cytokine, fold-change was calculated compared to mock-inoculated mice and $\log_2$ (fold-change) was plotted in the corresponding color-coded heat-map. (**B and D**) Cytokines were measured by multiplex platform in the lungs of SARS-CoV-2-infected mice after vaccination (n = 6 to 8 per group, two independent experiments). Mean values ± SEM are shown.
